# Self-Standing Adsorbent Composites of Waste-Derived Biochar and Reduced Graphene Oxide for Water Decontamination

**DOI:** 10.3390/molecules30091997

**Published:** 2025-04-30

**Authors:** Anna Dotti, Marianna Guagliano, Vittorio Ferretti di Castelferretto, Roberto Scotti, Simone Pedrazzi, Marco Puglia, Romano V. A. Orrù, Cinzia Cristiani, Elisabetta Finocchio, Andrea Basso Peressut, Saverio Latorrata

**Affiliations:** 1 Department of Chemistry, Materials and Chemical Engineering “Giulio Natta”, Politecnico di Milano, Piazza Leonardo Da Vinci 32, 20133 Milano, Italy; anna.dotti@polimi.it (A.D.); vittorioferrett@gmail.com (V.F.d.C.); cinzia.cristiani@polimi.it (C.C.); andreastefano.basso@polimi.it (A.B.P.); 2 Department of Organic Chemistry, Aachen-Maastricht Institute for Biobased Materials (AMIBM), Maastricht University, Urmonderbaan 22, 6167 KD Geleen, The Netherlands; r.orru@maastrichtuniversity.nl; 3 Department of Materials Science, INSTM, Università degli Studi di Milano Bicocca, Via R. Cozzi 55, 20125 Milano, Italy; roberto.scotti@unimib.it; 4 Department of Engineering “Enzo Ferrari”, Università degli Studi di Modena e Reggio Emilia, Via Vivarelli 10/1, 41125 Modena, Italy; simone.pedrazzi@unimore.it (S.P.); marco.puglia@unimore.it (M.P.); 5 Department of Civil, Chemical and Environmental Engineering—DICCA, Università di Genova (UniGe), Via All’Opera Pia 15, 16145 Genova, Italy; elisabetta.finocchio@unige.it

**Keywords:** biochar, reduced graphene oxide, freestanding membrane, self-assembling, heavy metals, adsorption, water treatment

## Abstract

Adsorption is one of the simplest and most cost-effective techniques for water decontamination. In this field, biochar has recently emerged as a promising alternative to traditional adsorbents, exhibiting a high surface area and affinity to metal ions, as well as often being waste-derived. Similarly, reduced graphene oxide (rGO) shows an excellent adsorption capacity. Having self-assembling properties, it has already been employed to obtain self-standing heavy-metal-adsorbing membranes. In this research, a novel self-standing membrane of biochar and rGO is presented. It was obtained through an eco-friendly method, consisting of the simple mechanical mixing of the two components, followed by vacuum filtration and mild drying. Vine pruning biochar (VBC) was employed in different rGO/biochar mass ratios, ranging from 1/1 to 1/9. The best compromise between membrane integrity and biochar content was achieved with a 4/6 proportion. This sample was also replicated using chestnut-shell-derived biochar. The composite rGO–biochar membranes were characterized through XRD, FTIR-ATR, TG-DTG, SEM-EDX, BET, ZP, particle dimension, and EPR analyses. Then, they were tested for metal ion adsorption with 10 mM Cu^2+^ and 100 mM Zn^2+^ aqueous solutions. The adsorption capacity of copper and zinc was found to be in the range of 1.51–4.03 mmol_Cu_ g^−1^ and 18.16–21.99 mmol_Zn_ g^−1^, respectively, at an acidic pH, room temperature, and contact time of 10 min. Interestingly, the composite rGO–biochar membranes exhibited a capture behavior between that of pure rGO and VBC.

## 1. Introduction

The release of heavy metals is a highly hazardous outcome of many human activities, spanning from industrial production (e.g., mining and smelting) to agriculture (e.g., fertilization) [1]. Soil represents the ultimate sink for the environmental accumulation of heavy metals, acting as the primary channel through which they spread to living organisms, the atmosphere, and water bodies [2]. Bioaccumulation in fruits and vegetables, drinking contaminated water, and direct ingestion or inhalation are the main pathways through which heavy metals enter the human body, posing a severe threat to human health [2,3,4,5]. Although some of these metal elements, such as Zn, Fe, Cu, and Cr, are necessary for the proper operation of certain biochemical functions, the accumulation of a few milligrams of these in the human body can become toxic [5]. An excessive ingestion of copper, for example, can lead to stomach, liver, and intestinal damage, as well as to skin cancer and angiosarcoma in the worst cases [2]. The accumulation of Cu^2+^ and Zn^2+^, in particular, is mainly caused by their use in the livestock industry. In particular, in pig farming, these metals are added to the animal feed, for example in the form of inorganic salts, zinc chelate of hydroxy analogue of methionine, and copper chelate of hydroxy analogue of methionine, to boost the content of minerals in their diet to improve their growth, and strengthen their immune system [1,6,7,8,9,10]. Although these metal ions are essential for the well-being of the animals, only about 10%, mainly for the inorganic salts, is adsorbed and the remaining part is excreted via excrement. Since a small amount of Cu^2+^ and Zn^2+^ administered is actually absorbed by the pigs, most of it ends up in their excrement and, accordingly, in the environment [11,12,13,14]. If pig manure is used as fertilizer, metal ions can bioaccumulate in soil, and when they are consequently adsorbed by crops, they enter the food chain and have adverse health effects on natural organisms and humans, such as permanent organ damage, in particular to the kidneys, and disruptions to the immune system [11,12,13,14].

Moreover, the Intermediate Dynamic Model for Metal (IDMM) [15] predicted that zinc tends to accumulate more in the acidic sandy soils than other soil types after the use of treated manure, showing a greater tendency to drain and runoff into surface water [10].

In this scenario, it is essential to identify a proper method to remove Cu^2+^ and Zn^2+^, preventing their spread into the environment [6,7].

Heavy metal removal can be carried out with conventional technologies, including chemical precipitation, ion exchange, adsorption, coagulation, and flocculation, as well as electrochemical processes, or with more recent strategies, such as biosorption or photocatalysis [16,17]. Among these, adsorption-based methods appear to be the most promising because of their low cost, high efficiency, simple operation, low consumption of solvents, high recovery efficiency, fast extraction time, and the possibility of regenerating sorbents [18,19,20]. An innovative and environmentally friendly adsorbent material is biochar (BC), the carbonaceous porous by-product of the residual biomass gasification process, characterized by an extraordinarily high porosity, large surface area, and high recalcitrance [21]. In addition, biochar shows a high affinity both with organic molecules [22,23,24,25,26] and heavy metals [18,27,28,29,30,31].

However, the practical application of biochar in wastewater treatment is currently limited [32] due to its low density and small particle size, which hinder its separation from water [33]. Being an effective adsorbent for contaminants, BC can act as an active carrier for pollutants. This results, through a co-transportation mechanism, in increased water and soil pollution [34]. Recovering the sorbent material still appears to be the most difficult step in the implementation of water treatment processes employing biochar [35]. In order to minimize environmental safety risks and to enable material reuse, biochar particles should be immobilized in macrostructures. Biochar-based membranes, in particular, appear as a promising strategy to limit the shedding and transport of fine BC particles, while preserving its excellent adsorption capability [36]. Mixed matrix membranes have been mainly developed by dispersing biochar particles as active fillers in a continuous phase. Typically, polymeric materials are used for the continuous phase, namely polyvinyl chloride (PVC) [37], polyvinylidene fluoride (PVDF) [38], polydopamine (PDA) [39], or polysulfone (PSF) [40]. However, biochar/polymer composite membranes may be subject to the common drawbacks of polymeric membranes, such as their swelling in solvents and decomposition at high temperatures and critical pH conditions, which would exclude many application fields [1]. Moreover, excluding the one proposed by Ghaffar et al. [38] and Saad et al. [41], all the above-mentioned membranes require a support material, e.g., glass.

Reduced graphene oxide (rGO) has attracted great interest for water purification applications in the last decade [42,43,44]. It can be effectively employed to capture organic molecules and metal ions [45,46]. Moreover, showing self-assembling properties, rGO can assemble in self-standing membranes, without compromising its adsorption capacity [47,48,49]. Coupling biochar (BC) with rGO presents multiple advantages. Compared to BC alone, rGO is responsible for an improvement of the adsorption performances in rGO–BC composites [50,51,52,53,54,55]. Furthermore, the self-assembling ability of rGO can be exploited to obtain self-standing rGO–BC objects [56,57].

The aim of this work is to obtain a self-assembling rGO–biochar membrane through a simple and eco-friendly process. For this purpose, different ratios between biochar and rGO were evaluated to find the best compromise between the maximization of biochar content and the preservation of the self-assembling properties of the membrane. Pristine materials and membranes were characterized by means of X-ray diffraction (XRD), scanning electron microscopy and energy-dispersive X-ray analysis (SEM-EDX), Fourier-transform infrared spectroscopy in attenuated total reflectance (FT-IR ATR), and thermogravimetric analysis (TG-DTG).

Moreover, aqueous solutions with Cu^2+^ and Zn^2+^, whose contents are similar to the real metal concentrations in piglets’ manure found in a rearing system in Northern Italy [11], have been tested to assess the metal ion removal properties of the proposed membranes. The Cu^2+^ adsorption was also evaluated by electron paramagnetic resonance (EPR) measurements.

Our innovative approach develops self-standing membranes composed of biochar, a sustainable and cost-effective material, and rGO, a well-known material with self-assembling and adsorption capabilities. Even though a combination of biochar and rGO has already been explored in the literature [58], to the best of our knowledge, this is the first study carried out to develop rGO–biochar freestanding membranes just by mixing rGO and biochar for wastewater treatment applications.

## 2. Results and Discussion

### 2.1. Membrane Preparation

**Figure 1:**
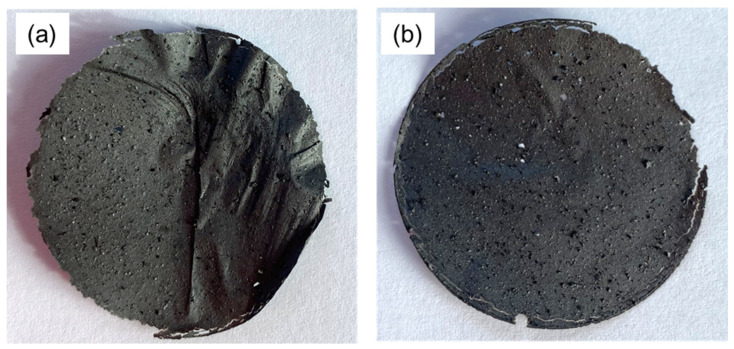
rGO–BC membranes with 60% biochar content: (**a**) vine pruning biochar (rGO–VBC) and (**b**) chestnut shell biochar (rGO–CBC).

Mixed membranes containing different amounts of reduced graphene oxide and vine pruning biochar were produced. Starting from 50% *w*/*w*, the vine pruning biochar (VBC) content was progressively increased to 60, 70, 80, and 90% *w*/*w*; samples representing the two limit conditions, i.e., 100% rGO and 100% VBC, were also prepared. The 100% VBC suspension did not allow the formation of a membrane, confirming that biochar has no self-assembly capability. Conversely, all the rGO-based suspensions were able to form a quite uniform membrane layer, which could be peeled off from the PVDF supporting membrane. Remarkably, even 10% rGO was enough to obtain a membrane with self-assembling capabilities. Nevertheless, the membranes were cracked and damaged for a VBC content equal to or higher than 70% and thus were difficult to handle. Samples with VBC contents of 50% and 60%, in contrast, were resistant enough to endure manipulations without cracking. Considering that the target was to replace rGO with the highest biochar content, the membrane with a mass ratio of rGO/VBC = 40/60 (rGO–VBC, Figure 1a) was considered to have the best result. Similarly, an intact and self-standing membrane was obtained by replacing 60% of rGO with chestnut-waste-derived biochar (rGO–CBC, Figure 1b). This suggests that the self-assembling properties of the system are not affected by the biochar’s characteristic, but are mainly dependent on the content of rGO, which is able to exert its assembly capability up to this mass ratio. Accordingly, rGO–VBC and rGO–CBC membranes were characterized and tested for metal adsorption.

### 2.2. Pristine Material and Membrane Characterization

In Table 1, the surface area (SA), particle dimensions, and zeta potential (ZP) of pristine VBC and CBC are reported.

The two biochars exhibit different properties (Table 1). CBC shows a very high surface area, more than one order of magnitude greater than the one of VBC. This may indicate the presence of undecomposed biomass in the VBC sample, which possibly resulted in the clogging of pores. The values for the VBC’s zeta potential support this picture. In fact, the ZP value has been related to the nature and the number of surface functional groups, meaning that a highly negative ZP corresponds to a large number of negatively charged groups, which are abundant in undecomposed biomass [59]. Moreover, the absolute value of ZP has been reported to increase when lowering the pyrolysis temperature [59]. Thus, the presence of undecomposed biomass in VBC is in accordance with its high ZP value (−39 mV). On this basis, it can be speculated that the VBC sample was obtained at a lower pyrolytic temperature than CBC.

**Figure 2:**
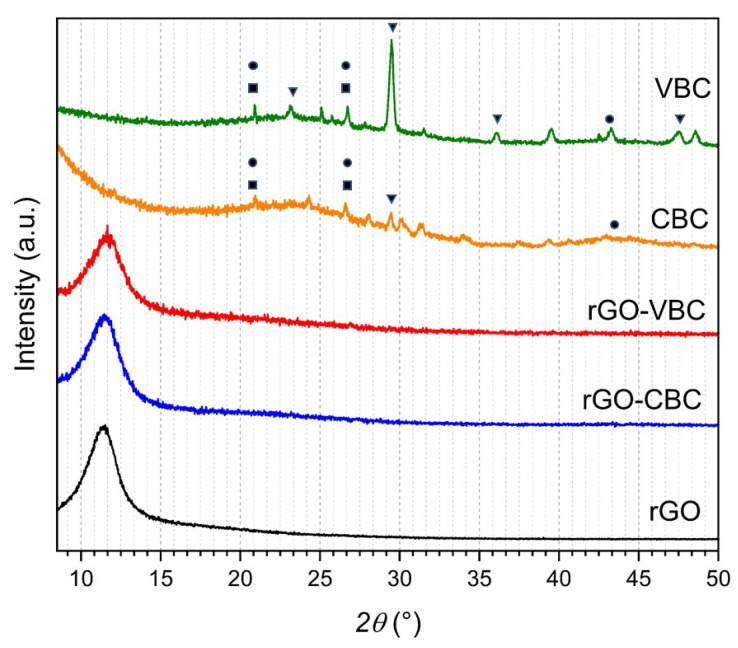
XRD patterns of pristine components and rGO–biochar mixed membranes (● graphite; ▼ calcite and dolomite; ■ hemicellulose and cellulose).

In Figure 2, the X-ray diffraction patterns the pristine materials and of the 40/60 rGO–VBC and rGO–CBC membranes are reported.

In both XRD patterns of the pristine biochars, the broad reflections centered between 22 and 26 2θ° and at about 43 2θ° are consistent with the presence of graphite layers, as a result of the biomass decomposition [60]. Furthermore, the reflections at 23, 29.4, 35.9, and 47.5 2θ°, which are stronger and better defined in VBC than in CBC, are consistent with the presence of a polycrystalline CaCO_3_ calcite-like phase containing impurities of Mg ions [61]. As a matter of fact, inorganic phases, such as calcite, are formed by the natural inorganic components, mainly Ca and Mg, of the biomass cells. Finally, the reflections at 22 and 26 2θ° could be attributed to residual cellulose and hemicellulose that partially overlap the graphite layers [60,62].

In the case of the rGO diffractogram, only the typical intense reflection at 11.3 2θ°, corresponding to the (001) plane of rGO [56], is detected. This reflection is the only one detected in the XRD patterns of the mixed membranes (11.6 and 11.5 2θ° for rGO–CBC and rGO–VBC, respectively), and no reflections related to the calcite or graphite phases are detected. This finding may be ascribed to the lower content of biochar (60%) in the mixed membranes, compared with pristine biochar, which make calcite and graphite detection improbable.

**Figure 3:**
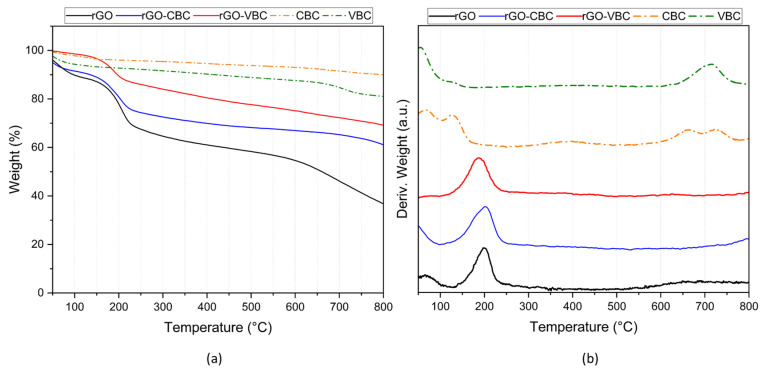
(**a**) TG and (**b**) DTG curves of pristine biochar and of rGO–biochar mixed membranes.

The thermograms obtained from the TGA of the nitrogen of the pristine biochars (VBC and CBC), the rGO, and the corresponding membranes (rGO–VBC and rGO–CBC) are compared in Figure 3. Biochar thermograms are characterized by a continuous weight loss of about 10–20% (Figure 3a). In contrast, the decomposition curve of the pristine rGO shows a high weight loss, about 70%, characterized by three steps.

The thermal decomposition features of the mixed membranes are intermediate between those of rGO and biochars. The thermal decomposition behavior of the samples, especially in the case of the mixed membranes, can be better analyzed by DTG, as shown in Figure 3b, in which thermal phenomena are highlighted. The DTG results can be summarized as follows: (i) the thermal phenomenon, in the range of 30–100 °C, which is common across all the samples, is associated with the evolution of physiosorbed water molecules; (ii) the strong phenomenon between 150 and 250 °C may account for the decomposition of the residues of ascorbic acid and oxygenated moieties present in rGO [47,63]; (iii) the broad and barely visible phenomenon occurring above 600 °C can be ascribed to the degradation of the graphitic framework; (iv) finally, the phenomenon in the range of 650–750 °C may correspond to the thermal decomposition of traces of the calcite phase in biochars [64].

**Figure 4:**
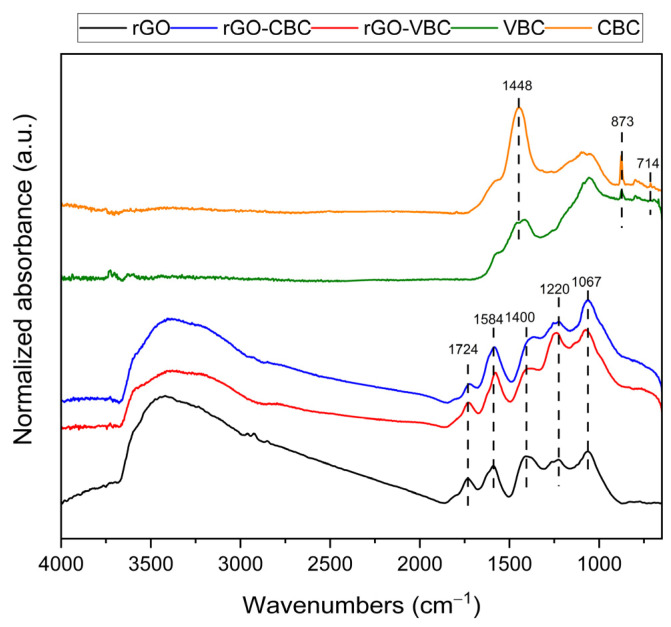
FT-IR ATR spectra of pristine components and rGO–biochar mixed membranes.

The pristine components and mixed membranes were also analyzed by FT-IR spectroscopy in ATR mode (Figure 4).

The pristine biochars exhibit very similar spectra, characterized by a main band centered around 1448 cm^−1^, accompanied by a sharp band at 873 cm^−1^, and weak components near 714 cm^−1^, which are characteristic of carbonate species [65]. The broad envelope of bands in the spectral region of 1200–1000 cm^−1^ can be due to the overlapped CC and CO stretching modes, together with ring vibration and COH deformation, assigned to the residual cellulosic material. These findings also support the results of the XRD analysis [66].

In contrast, the spectrum of rGO is characterized by the presence of several bands, all of them consistent with those reported in the literature for graphene oxide [59]. The reduction treatment by L-AA leads indeed to a significant decrease in the intensity of these features, which are due to residual oxygen-containing groups. Specifically, the bands centered at 1067 and 1220 cm^−1^ are associated with the C-O stretching vibrations of residual hydroxyl and alkoxy groups [67]. The contributions at about 1400, 1584, and 1724 cm^−1^ might refer to, respectively, the C–H bending of alkyl chains and/or O–H deformation modes of carboxylic groups, C=C stretching in graphene oxide layers, and C=O stretching in carboxylic and carbonyl groups [47,68]. Finally, the broad and strong absorption band between roughly 3000 and 3500 cm^−1^ may be attributed to O–H stretching modes [47,67,68].

The bands detected in the spectra of the composite membranes are consistent with those of rGO, while the biochar features are no longer clearly detected. It can be speculated that the biochar features overlap those of rGO, with the latter being quite intense. This would explain the general increase in the region of 1250–1000 cm^−1^. The absence of carbonate species is likely due either to their dissolution in the slightly acidic, reduced GO suspension during the preparation procedure or to a possible inhomogeneous distribution in the analyzed membrane portion.

**Figure 5:**
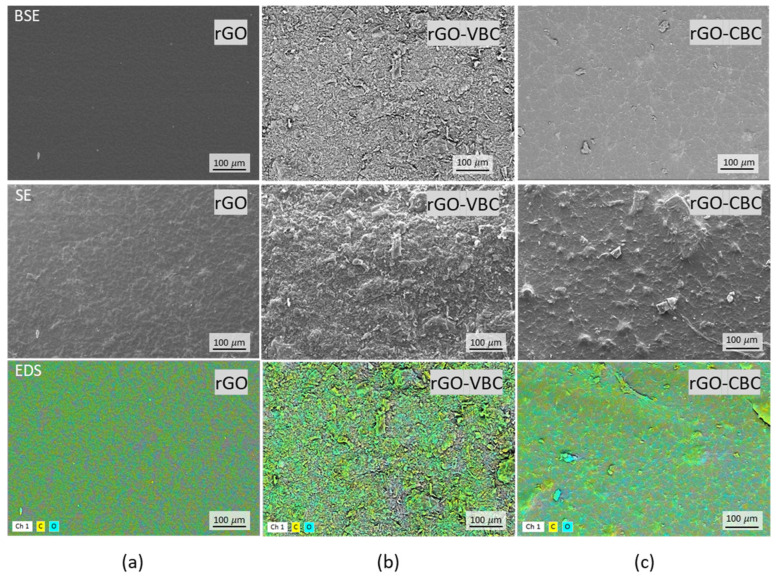
SEM-EDX images at 500× magnification of (**a**) rGO, (**b**) rGO–VBC, and (**c**) rGO–CBC. Backscattered electrons (**top**), secondary electrons (**middle**), and EDX maps indicating carbon in yellow and oxygen in light blue (**bottom**).

SEM images from backscattered electrons, secondary electrons, and SEM-EDX analyses of the rGO, rGO–VBC, and rGO–CBC membranes are reported in Figure 5a–c.

The surface of the rGO membrane (Figure 5a, top and middle) appears as a continuous, flat, and homogeneous layer, without distinguishable cracks or aggregates. Carbon and oxygen are uniformly distributed on the surface (Figure 5a, bottom). Conversely, the surface of both the mixed rGO–VBC and rGO–CBC membranes (Figure 5b,c) is characterized by an irregular morphology, in which the presence of aggregates, although apparently incorporated in a matrix, is manifest (Figure 5b,c, top and middle). Aggregates are much more appreciable in the case of the rGO–VBC membrane (Figure 5b), whose surface morphology and elemental distribution in the EDX map (Figure 5b, bottom) appear to be more heterogeneous. The presence of a continuous matrix incorporating the aggregates is consistent with the self-assembly effect exerted by rGO, which apparently is not perturbed by the presence of biochar. In this respect, it has to be noted that the pristine biochars, both CBC and VBC, are not able to form freestanding films.

### 2.3. Cu^2+^ and Zn^2+^ Adsorption Tests

rGO–VBC membranes with a 60% content of biochar, being selected as the best compromise between maximizing VBC and sample resistance to manipulation, were employed in Cu^2+^ and Zn^2+^ adsorption tests in mono-ionic aqueous solutions. Pristine rGO and VBC were also tested for comparison. Each sample was put in contact with a 50 mL solution containing either 62.5 mmol g^−1^ of Cu^2+^ or 625.0 mmol g^−1^ of Zn^2+^. The solutions’ compositions were selected to simulate real ion concentrations in weaned pigs sludge [11].

The specific captured amount of metal ions Q_m_ (mmol g^−1^) for each sample was obtained via Equation (2). Moreover, the theoretical adsorption capacity Q_m,th_ for the rGO–VBC mixed membrane was calculated according to Equation (1), considering the experimental Q_m_ values of rGO and VBC.

(1) $$Q_{m,th}\left(\mathrm{rGO}-\mathrm{VBC}\right)=0.40\cdot Q_{m}\left(rGO\right)+0.60\cdot Q_{m}(VBC)$$

Experimental and theoretical capacities are summarized in Table 2.

All the sorbents show a low sorption efficiency, in the range of 3–6%, and rGO–VBC exhibits an intermediate level of adsorption between the levels of the pristine components. The low capture percentages are apparently far from those reported in the literature, with the initial concentrations selected for these experiments (Cu^2+^ 10 mM and Zn^2+^ 100 mM) being considerably higher than those applied in previous studies (in the range of 0.1–3 mM) [69,70,71]. Also, the particular testing setup employed in this research makes comparisons of efficiency with other studies difficult and not very representative.

During the experiments, the pH values of the solutions were measured before and after the adsorption tests (Table 3).

It is evident that the solution pH during the contacting experiment is driven by the solid sorbent; indeed, before the tests, it is weakly acidic, in the range of 4.6–4.9. Once the solutions are put in contact with the sorbent, the sorbent nature modifies the pH in view of the acidity/basicity of the surface. It can be hypothesized that, in the case of rGO, the pH strongly decreased in view of the presence of the residual amount of L-ascorbic acid used for the reduction, which is released during the tests. In contrast, the slight pH increase observed for VBC can be explained by the presence of the residual amount of calcite. Indeed, nitrates present in the contacting solution are able to displace calcite carbonates, so that calcium ions’ dissolution induces the slight pH increase. In the case of the mixed membrane, the acidification due to the rGO component is predominant, in particular for Cu^2+^. This last observation could be related either to slight differences in the membrane composition, as the two tests have been performed on two different membranes, or to the higher basicity of Zn ions.

The low capture capability of the mixed membrane does not seem to be caused by the poor stability of the membrane. Indeed, as reported in the literature, rGO membranes tend to remain stable over time and in different pH conditions [72,73,74,75]. Biochar is considered to be a stable material as well. However, its stability may vary with other properties, such as its surface area, pH, and porosity. A method to assess biochar’s stability involves the evaluation of its aromatic characteristics, as shown in both VBC and CBC by XRD analysis, which usually become more pronounced at higher pyrolysis temperatures [76,77]. Furthermore, research concerning the combination of rGO and biochar has demonstrated the formation of stable materials [41,78].

Conversely, the low capture capability of the mixed membrane can be justified considering the membrane’s composition, i.e., 60% *w*/*w* of VBC and 40% *w*/*w* of rGO. As a matter of fact, the theoretical adsorption capacity of the mixed membrane, being the combination of those of the single components, is negatively affected by the limited rGO content, i.e., the most efficient sorbent (Table 2).

However, the actual adsorptions Q_m_ of rGO–VBC, 2.19 for Cu^2+^ and 19.12 mmol g^−1^ for Zn^2+^, are consistent with Q_m,th_, i.e., 2.52 mmol g^−1^ and 20.46 mmol g^−1^ for Cu^2+^ and Zn^2+^, respectively. This may suggest that neither synergistic nor antagonistic effects are present, and the adsorption capacity of rGO–VBC is just the weighted sum of the capacities of the single components. Therefore, despite the thorough mixing observed by SEM-EDX (Figure 5b), the components are just mixed and, apparently, they do not interact with each other.

**Figure 6:**
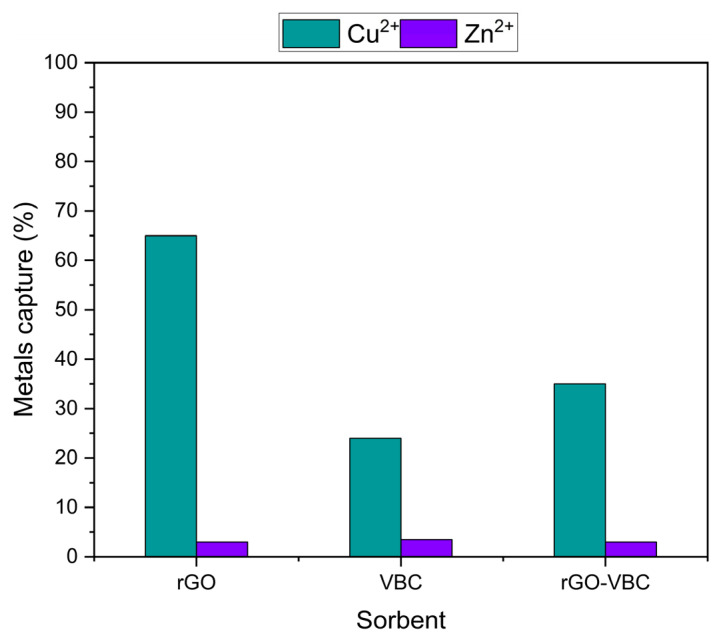
Metal capture efficiency of the different sorbents.

The capture yield of the different sorbents has been calculated and is plotted in Figure 6.

Comparing the Zn and Cu yield results, it is evident that Cu capture is much more efficient than Zn capture for all the sorbents. The best Cu capture is obtained by the use of rGO (65%), and it is only partially preserved in the mixed membrane. Instead, a very low and constant Zn capture (about 3%) was found. It can be argued that all the sorbents possess a higher affinity for Cu than for Zn, but it has to be noted that the initial Zn content is two orders of magnitude higher than the Cu content (6.25 and 625 mmol g^−1^ for Cu and Zn, respectively). Furthermore, the constant Zn adsorption suggests the presence of a site saturation effect, but all these considerations need to be confirmed in further studies with solutions at different initial concentrations. However, the presence of a selectivity effect is of paramount importance when the two ions are co-present in solution, as in the case of real sludges.

Moreover, although the study of adsorption isotherms is not the primary focus of this work, we expect that the materials could demonstrate behavior consistent with Langmuir and Freundlich adsorption isotherms, as reported in the literature [52,70,79,80].

**Figure 7:**
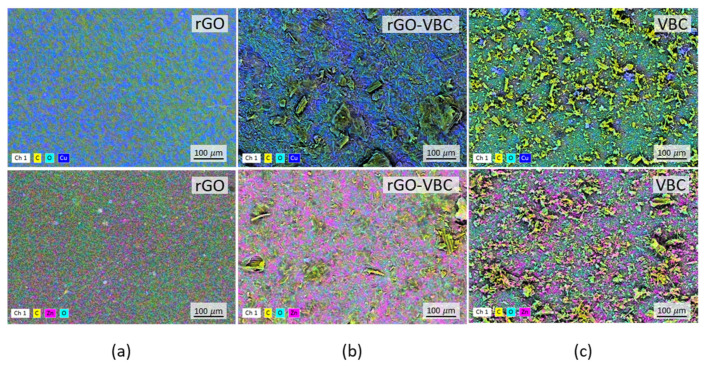
SEM-EDX elemental maps at 500× magnification of (**a**) rGO, (**b**) rGO–VBC, and (**c**) VBC after adsorption of Cu^2+^ (**top**, blue spots) and Zn^2+^ (**bottom**, pink spots).

The membranes after the adsorption experiments were characterized by means of SEM-EDX spectroscopy (Figure 7a–c). The images confirm the presence of Cu^2+^ (Figure 7a–c, top, blue spots) and Zn^2+^ (Figure 7a–c, bottom, pink spots) on the sorbents’ surface. Cu is uniformly distributed onto the rGO membrane (Figure 7a, top), while is apparently localized in specific areas in the case of the pristine VBC (Figure 7c, top). Similarly, for Zn^2+^ ions, the metal is quite homogeneously distributed on the rGO membrane, but it is clearly adsorbed in specific areas in the VBC (Figure 7c, bottom). The mixed rGO–VBC membranes again feature an intermediate situation (Figure 7b), in which the homogeneous distribution of the ions coexists with local accumulation and clustering. This is particularly evident in the case of Zn^2+^ (Figure 7b, bottom).

Analyzing the EDX maps, it is hard to find a correlation between functional groups, namely the oxygenated ones, which are presumably those capable of interacting with the ions, and adsorption sites. Indeed, as shown in the maps, the light blue spots, corresponding to oxygen, are homogeneously spread on the surface of all the solids, and no correlation between the surface area elemental composition and the formation of adsorbed ions’ clusters is visible.

**Figure 8:**
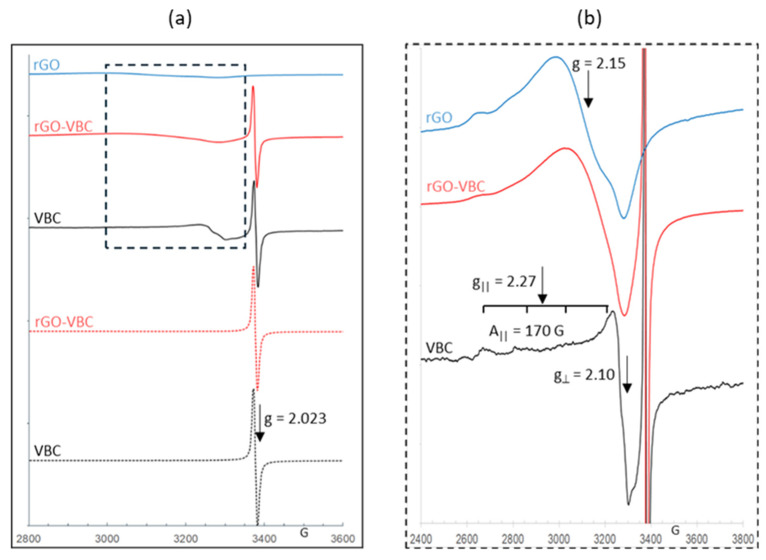
(**a**) EPR spectra of sorbents before (dotted lines) and after (solid lines) Cu^2+^ adsorption tests; (**b**) magnification, corresponding to the dashed line box, of Cu^2+^ spectra.

Considering the difficulty of obtaining information on the adsorption sites, the membranes after copper adsorption were also evaluated by electron paramagnetic resonance (EPR) spectroscopy. The EPR spectra of VBC in powder form as well as of rGO–VBC and rGO membranes after the adsorption of a 10 mM solution of Cu^2+^ are reported in Figure 8, comparing them with the spectra of the bare biochar powder and the as-prepared rGO–VBC membrane as a blank. The spectra of all samples containing biochar show typical isotropic signals at g = 2.023 due to the carbon-centered permanent free radicals (PFR) of biochar [81,82], either before or after Cu adsorption. After the adsorption of copper, the EPR spectra of all the membranes show the presence of Cu^2+^ species, with an intensity which confirms the trend of copper adsorption obtained by ICP-OES analysis. In particular, the copper EPR signal in VBC powder has the lowest intensity compared to those of the rGO–VBC and rGO membranes. The adsorbed Cu signal exhibits values of g|| = 2.27, g_^_ = 2.10, and resolved parallel hyperfine structures A|| = 170 G, which can be assigned to Cu^2+^ centers with tetragonally distorted octahedral symmetry [83]. On the other hand, the EPR copper spectra of both rGO–VBC and rGO are characterized by the overlapping of Cu^2+^ center signals similar to those in VBC powder and by a broad and unstructured line centered at g ≈ 2.15, which is typical of dipolar coupled Cu^2+^ species with a disordered coordination environment and is likely related to the highest amount of copper adsorbed by the two membranes [84].

## 3. Materials and Methods

### 3.1. Materials

A commercial biochar (VBC), produced via a proprietary process from vine pruning biomass, was supplied by Romagna Carbone (Bagnacavallo, RA, Italy). Chestnut biochar (CBC) was prepared at the BEELab of Modena and Reggio Emilia University by a gasification process (with maximum temperature of 800–1000 °C) using chestnut shells and pericarps (provided by Az. Agr. Agriappennino di Sepe Marco, Cecciola, RE, Italy) as feed biomass. In particular, it was pelletized to be used as fuel in a fixed-bed downdraft gasifier prototype. It has an Imbert-throated hearth reactor designed to operate with a biomass flow of a few kg h^−1^, making it ideal for testing when only a limited amount of fuel is available [85]. Graphene oxide in the form of a 4 mg mL^−1^ aqueous dispersion (particle size < 10 µm) was acquired from Graphenea (San Sebastian, Spain). L-ascorbic acid (L-AA), Cu(NO_3_)_2_∙3H_2_O, and Zn(NO_3_)_2_∙6H_2_O were supplied by Sigma-Aldrich (Milan, Italy).

### 3.2. Membranes Preparation

**Figure 9:**
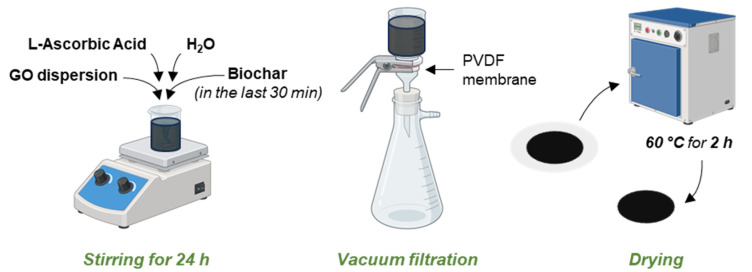
Membrane preparation procedure.

Membranes were prepared according to a simple previously developed protocol [47,86], which was adapted for the addition of biochar (Figure 9). In particular, the GO commercial dispersion, after dilution to 0.4 mg mL^−1^ and ultrasonication (Labsonic LBS1-6, Falc Instruments, Faenza, Italy), was reduced with L-AA (L-AA:GO = 10:1 by weight). The suspension was stirred for 24 h at room temperature. Biochar, previously ground in a mortar until obtaining a uniform and fine powder, was added to the above suspension 30 min before the end of the stirring time. The mixture was then poured in a Gooch crucible and vacuum-filtered onto a polyvinylidene fluoride (PVDF) membrane with a 47 mm diameter and 0.45 µm pore size (Merck Millipore, Burlington, MA, USA). The resulting membranes were dried in an oven for 2 h at 60 °C.

Setting a total membrane weight of 8 mg, different rGO/BC ratios were considered; namely, biochar was introduced to replace 50, 60, 70, 80, and 90% *w*/*w* rGO. Pure biochar and pure rGO samples were also prepared to be used as reference. Specifically, for the pure rGO membrane, the above-mentioned conventional procedure was applied, without the addition of BC. Conversely, for the pure biochar sample, 8 mg of BC was dispersed in H_2_O, then it was vacuum-filtered and dried as previously described.

### 3.3. Pristine Materials and Membranes Characterization

Surface area (SA) was measured by performing Brunauer–Emmett–Teller (BET) analysis (in N_2_ atmosphere and bath temperature of 77.35 K) with the ASAP 2420 apparatus by Micromeritics (Norcross, GA, USA) on samples previously outgassed at 120 °C to a residual pressure of <10^−4^ Pa.

X-ray diffraction (XRD) patterns of the pristine powders were collected at room temperature in the range of 8–50° 2θ through a D2-Phaser diffractometer (Bruker Italy, Milan, Italy), equipped with Cu radiation (λ = 1.54 Å) using Bragg Brentano geometry.

Particles’ dimensions were determined by laser granulometry (CILAS 1180 instrument, Orleans, France) according to Klank et al. [87].

Zeta potential (ZP) was determined using 5 mg L^−1^ of biochar dispersed in water, at the pH of the solid in water, using a Zetasizer Nano ZS (Malvern Instruments Limited, Malvern, UK). Dynamic light scattering (DLS) at 90°, with non-invasive backscatter (NIBS) optics, was used for measurements.

Macro-Fourier-transform infrared spectroscopy in attenuated total reflectance (ATR FT-IR) spectra were recorded using an FTIR Nicolet Nexus spectrometer, which was equipped with a single reflection silicon crystal, a mercury cadmium telluride (MCT) detector cooled with liquid nitrogen, and a ThermoElectron Continuμm IR microscope, all supplied by Thermo Fisher Scientific Inc. (Rodano, Italy). Spectra were collected in the 650–4000 cm^−1^ range with 128 scans and a spectral resolution of 4 cm^−1^.

Thermogravimetric analysis (TG-DTG) from room temperature up to 850 °C was performed using a TGAQ500 (TA Instruments, Eschbor, Germany) with a heating rate of 10 °C min^−1^ in nitrogen atmosphere.

Scanning electron microscopy and energy-dispersive X-ray spectroscopy (SEM-EDX), before and after Cu^2+^ and Zn^2+^ capture tests, were performed using a Zeiss EVO 50 EP (Zeiss, Jena, Germany) equipped with an INCA energy 2000 spectrometer (Oxford Instruments, Abingdon-on-Thames, UK), operated at an electron high tension (EHT) voltage of 20 kV, with a current probe of 100 pA, and in high vacuum mode (10^−4^ Pa).

Electron paramagnetic resonance (EPR) measurements were performed before and after Cu^2+^ adsorption at 123 K using an EMX spectrometer (Bruker Italy, Milan, Italy) operating at the X-band frequency and equipped with an Oxford Instruments cryostat (Abingdon-on-Thames, UK). Spectra were recorded at a power of 2 mW, modulation amplitude 2 or 10 G, and at 130 K.

### 3.4. Adsorption Tests

For metal ions’ adsorption tests, mono-ionic aqueous solutions of Cu^2+^ 10 mM (635 mg L^−1^) and Zn^2+^ 100 mM (6530 mg L^−1^), which are close to the real concentration values in piglets’ manure [11], were prepared. Exploiting the same filtration setup reported in Figure 9, the adsorption experiments were carried out by putting each membrane in contact with 50 mL of mono-ionic solution for 10 min, which was poured into the Gooch crucible. Then, the solution was vacuum-filtered through the membrane. The pH values of the solutions, both as-prepared and after the contacting process, were measured (FiveEasy pH-meter equipped with an LE438 electrode, Mettler Toledo, Columbus, OH, USA), but no pH correction was applied.

Metal ions’ concentration in solution was determined before (C_i_) and after (C_f_) the adsorption tests by means of inductively coupled plasma optical emission spectroscopy (ICP-OES) using an OPTIMA 7000 DV spectrometer (PerkinElmer, Waltham, MA, USA). Reported values are the average of three measurements, with an estimated error of 2.5%. The specific captured amount of metal ions Q_m_ (mmol g^−1^) was obtained according to Equation (2), where C_i_ (mg L^−1^) and C_f_ (mg L^−1^) are the initial and final ion concentrations, respectively, V is the volume of the contacted solution (50 mL), AW is the atomic weight of copper or zinc, and w_membrane_ is the membrane weight (8 mg).

(2) $$Q_{m}\left(mmol\;g^{-1}\right)=\frac{(C_{i}-C_{f})\cdot V}{AW\cdot w_{membrane}}$$

## 4. Conclusions

The membrane preparation procedure here proposed represents an innovative, simple, and low-environmental-impact strategy to immobilize biochar for water treatment applications. Indeed, in this study, the self-assembling properties of reduced graphene oxide were exploited to immobilize waste-derived biochar in a self-standing membrane. Different compositions were explored, considering a biochar mass percentage from 50 up to 90%. In all cases, self-standing membranes were successfully obtained, but the sample composed of 60% BC and 40% rGO showed the best compromise between membrane resistance and maximizing the biochar content, and this result was effectively obtained by employing both vine pruning and chestnut shell waste-derived biochars.

The mixed rGO–VBC membrane, when tested for water decontamination from Cu^2+^ and Zn^2+^ ions, showed intermediate adsorption properties (2.19 mmol g^−1^ for Cu^2+^ and 19.12 mmol g^−1^ for Zn^2+^) between those of the two pristine components, which somewhat correspond to the theoretical one, calculated considering the mixed membrane composition.

Neither synergistic nor antagonistic effects on the capture capability are present in the mixed membrane, and the adsorption capacity of rGO–VBC is just the weighted sum of the capacities of the single components. Therefore, despite the thorough mixing observed by SEM-EDX, the components seem to be just mixed, with no apparent interactions between them.

A higher affinity for Cu than for Zn was found. The presence of a selectivity effect is crucial when the two ions are co-present in solutions, as occurs in real sludges. Thus, despite being preliminary, in the authors’ opinion, this work represents a step forward for the technological valorization of waste-biomass-derived materials.

## Figures and Tables

**Table 1:** Surface area, particle dimensions, and zeta potential of vine pruning (VBC) and chestnut shell (CBC) biochar (* pH of biochar in distilled water).

Sample	SA (m^2^ g^−1^)	Particle Dimensions d_10_, d_50_, d_90_ (µm)	Zeta Potential (mV)
VBC	28	6, 31, 66	−39 (* pH 8)
CBC	327	2, 25, 64	−6 (* pH 11)

**Table 2:** Cu^2+^ and Zn^2+^ capture (Q_m_) by VBC as function of the initial ions’ concentrations (* Q_m,th_ theoretical adsorption capacity via Equation (2)).

Sorbent	Cu^2+^			Zn^2+^		
	Initial Cu^2+^ (mmol g^−1^)	Q_m_ (mmol g^−1^)	Q_m,th_ * (mmol g^−1^)	Initial Zn^2+^ (mmol g^−1^)	Q_m_ (mmol g^−1^)	Q_m,th_ * (mmol g^−1^)
rGO	62.5	4.03	-	625.0	18.16	-
VBC		1.51	-		21.99	-
rGO–VBC		2.19	2.52		19.12	20.46

**Table 3:** pH values of Cu^2+^ and Zn^2+^ solutions before and after the filtration tests.

Ion	pH Before Tests	pH After Test		
		rGO	VBC	rGO−VBC
Cu^2+^	4.6	2.9	4.8	2.9
Zn^2+^	4.9	2.9	5.4	3.8

## Data Availability

The data presented in this study are available on request from the corresponding authors.

## Abbreviations

The following abbreviations are used in this manuscript:

BC	Biochar
CBC	Chestnut biochar
GO	Graphene oxide
L-AA	L-Ascorbic acid
rGO	Reduced graphene oxide
rGO–BC	Membrane composed of 40% rGO and 60% BC
rGO–CBC	Membrane composed of 40% rGO and 60% CBC
rGO–VBC	Membrane composed of 40% rGO and 60% VBC
SA	Surface area
VBC	Vine pruning biochar
ZP	Zeta potential
